# Plant non-coding RNAs function in pollen development and male sterility

**DOI:** 10.3389/fpls.2023.1109941

**Published:** 2023-02-15

**Authors:** Hushuai Nie, Cheng Cheng, Jie Kong, Huijing Li, Jinping Hua

**Affiliations:** ^1^ Agricultural College, Inner Mongolia Agricultural University, Hohhot, China; ^2^ Laboratory of Cotton Genetics, Genomics and Breeding/Key Laboratory of Crop Heterosis and Utilization of Ministry of Education/Beijing Key Laboratory of Crop Genetic Improvement, College of Agronomy and Biotechnology, China Agricultural University, Beijing, China; ^3^ Institute of Economic Crops, Xinjiang Academy of Agricultural Sciences, Urumqi, Xinjiang, China

**Keywords:** microRNA (miRNA), long non-coding RNA (lncRNA), plant hormone, mitochondrial gene, male sterility

## Abstract

Male sterility is classified as either cytoplasmic male sterility (CMS) or genic male sterility (GMS). Generally, CMS involves mitochondrial genomes interacting with the nuclear genome, while GMS is caused by nuclear genes alone. Male sterility is regulated by multilevel mechanisms in which non-coding RNAs (ncRNAs), including microRNAs (miRNAs), long non-coding RNAs (lncRNAs), and phased small interfering RNAs (phasiRNAs), which have been proven to be critical elements. The development of high-throughput sequencing technology offers new opportunities to evaluate the genetic mechanism of ncRNAs in plant male sterility. In this review, we summarize the critical ncRNAs that regulate gene expression in ways dependent on or independent of hormones, which involve the differentiation of the stamen primordia, degradation of the tapetum, formation of microspores, and the release of pollen. In addition, the key mechanisms of the miRNA–lncRNA–mRNA interaction networks mediating male sterility in plants are elaborated. We present a different perspective on exploring the ncRNA-mediated regulatory pathways that control CMS in plants and create male-sterile lines through hormones or genome editing. A refined understanding of the ncRNA regulatory mechanisms in plant male sterility for the development of new sterile lines would be conducive to improve hybridization breeding.

## Introduction

Heterosis has been utilized to increase productivity in many crops. The male sterility system provides crucial breeding tools to harness heterosis due to its biosafety and high efficiency and because it is less labor-intensive and time-consuming (Parodi and Gaju, 2009; Chen and Liu, 2014). Generally, the male reproductive development in plants comprises a series of stages of specification of the stamen primordia, generation of sporogenous cells, development of the tapetum and microspore mother cells, meiosis, formation of free haploid microspores, degeneration of the tapetum, and the release of mature pollen grains. The arrest of any of these steps could result in male sterility (Huang et al., 2014; Bohra et al., 2021; Han et al., 2021; Zhang et al., 2021). Plant male sterility refers to plants’ normal male organs failing to produce functional microspores, pollen, or anthers, but their pistils still having adequate flowering and crossing abilities with other plants (Parodi and Gaju, 2009; Brownfield, 2021). Male sterility, which was first observed by the German botanist Joseph Gottlieb Kolreuter in 1763 and has now been reported in hundreds of plant species (Laser and Lersten, 1972; Mayr, 1986; Kaul, 1988), can be classified as either cytoplasmic male sterility (CMS) or genic male sterility (GMS) (Zhu et al., 2020). Generally, CMS is ascribed to the uncoordinated inheritance between the organellar (mitochondrial, MT) and nuclear genomes, while GMS is caused by nuclear genes independently (Bohra et al., 2016).

**Figure 1:**
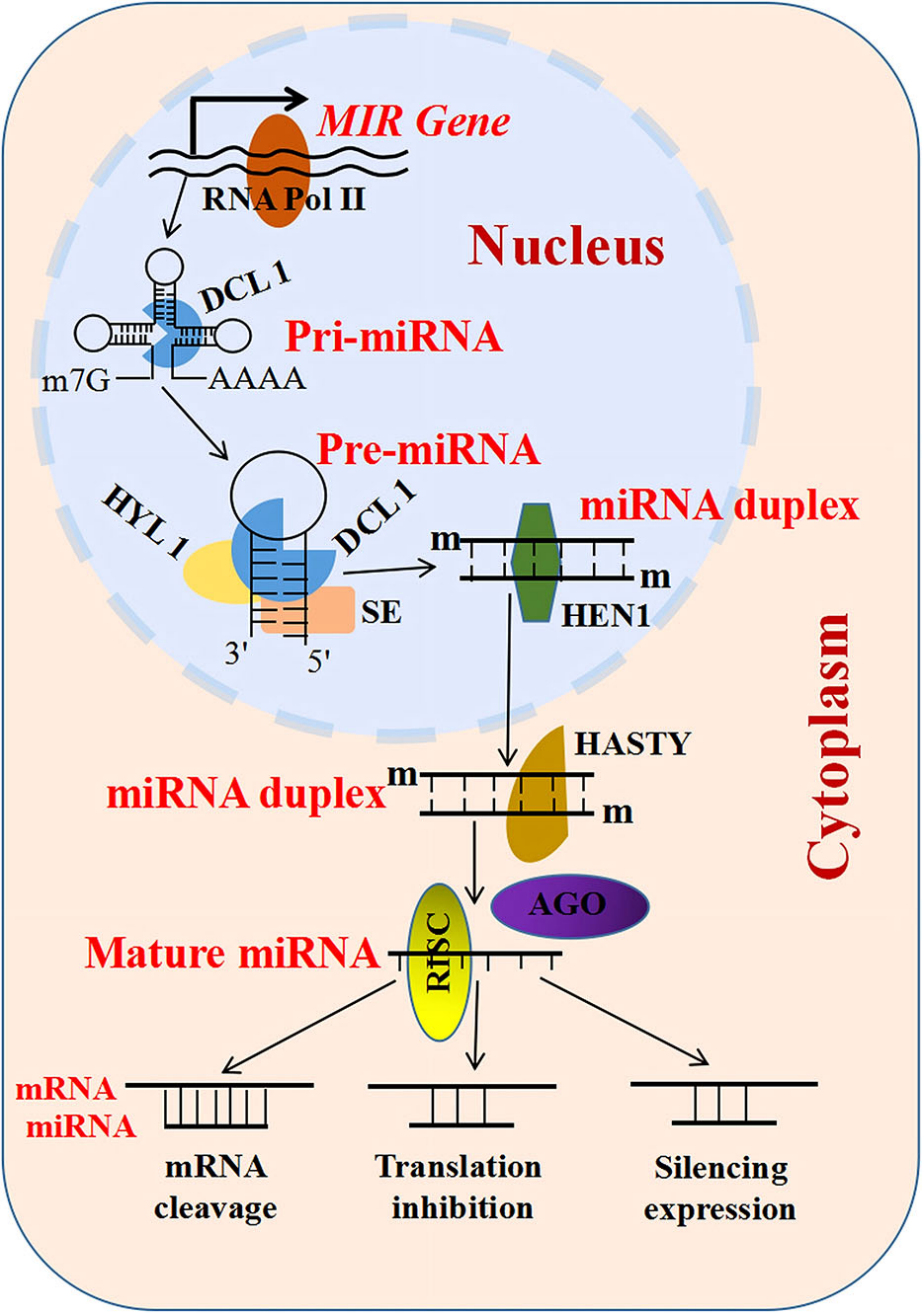
Characteristic features of microRNAs (miRNAs) and long non-coding RNAs (lncRNAs). Biosynthesis and mode of action of miRNAs: primary miRNAs (pri-miRNAs) self-fold and are complementarily paired to form one or more stable stem–loop structures, and are then processed by the ribonuclease III-like enzyme and DICER-LIKE 1 (DCL1) to generate a small hairpin-shaped RNA (precursor miRNA, pre-miRNA). The pre-miRNAs are further processed by DCL1 to produce a mature miRNA duplex. In this process, two proteins, HYPONASTIC LEAVES 1 (HYL1) and SERRATE (SE), also play important roles in the association with DCL1. HYL1 is a nuclear double-stranded RNA-binding protein, acting in the DCL1 complex to regulate the first step of the pri-miRNA process. SE encodes a C2H2 zinc finger protein, which is located in the nucleus and physically interacts with HYL1. *RISC*, RNA-induced silencing complex.

**Figure 2:**
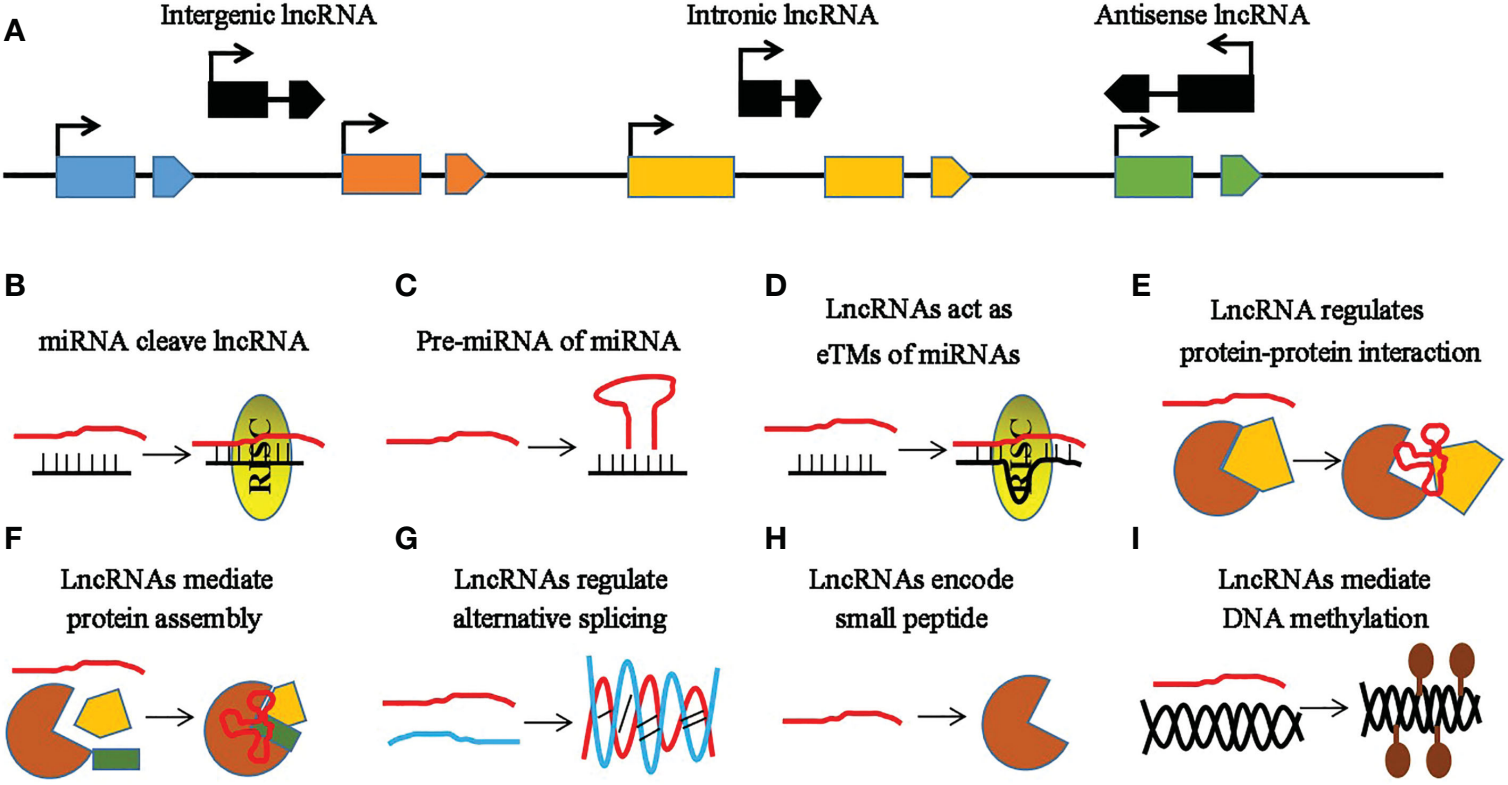
Characteristic features of long non-coding RNAs (lncRNAs). **(A)** Categories of lncRNAs based on their locations. The *black line* represents the chromosome, while the *rectangles* and *polygons with similar colors* represent the different exons of the protein-coding genes or lncRNAs. The *blue*, *brown*, *yellow*, and *green rectangles* and *polygons* represent different genes, while the *black rectangles* and *polygons* represent different lncRNAs. *Intergenic*, *intronic*, and *antisense* lncRNAs are the lncRNAs located outside the protein-coding genes, in the intron of a protein-coding gene, and those located in the antisense orientation to a protein-coding gene, respectively. *Arrows* indicate the direction of the gene or lncRNA. **(B–I)** Functional mechanisms of lncRNAs. The *red line* indicates the lncRNA, the *black line* represents the microRNA (miRNA), and the *brown semicircle* and *yellow diagram* indicate the proteins. **(B)** LncRNAs act as targets of miRNAs. **(C)** LncRNAs as precursor miRNAs (pre-miRNAs) of miRNAs. **(D)** LncRNAs as target mimics of miRNAs. **(E)** LncRNAs regulate protein–protein interactions. **(F)** LncRNAs as scaffolds regulating the assembly of protein complex subunits. **(G)** LncRNAs paired with their targeted RNAs. **(H)** LncRNAs encode small peptides. **(I)** LncRNAs contribute to epigenetic silencing *via* RNA-directed DNA methylation (RdDM).

Non-coding RNAs (ncRNAs) are important regulators of gene expression involved in various metabolisms (Xia et al., 2019; Chao et al., 2022) that are classified into three types: small ncRNAs [sncRNAs; 20–50 nucleotides (nt)], intermediate-sized ncRNAs (50–200 nt), and long ncRNAs (lncRNAs; >200 nt) (Hajieghrari and Farrokhi, 2022). MicroRNAs (miRNAs) comprise a well-studied subset of sncRNAs (Reinhart et al., 2002) whose biogenesis begins with the MIR genes (genes encoding miRNAs) inside the nucleus (**Figure 1**) (Guleria et al., 2011). Subsequently, these MIR genes are processed to form primary miRNAs (pri-miRNAs) and precursor miRNAs (pre-miRNAs) mediated by RNA polymerase II (RNA Pol II), ribonuclease III-like enzyme, and DICER-LIKE 1 (*DCL1*) (Guleria et al., 2011). Pre-miRNAs are small hairpin-shaped RNAs that are further processed by *DCL1* to produce a mature miRNA duplex. This miRNA duplex contains miRNA and miRNA^*^ strands. After transportation into the cytoplasm, the mature miRNA strand is loaded into the AGO (Argonaute) protein and forms a functional RNA-induced silencing complex (RISC) (**Figure 1**) (Wahid et al., 2010; Zhang et al., 2022a). Based on the degree of the miRNA–messenger RNA (mRNA) complementarity, miRNAs primarily mediate gene regulation in three different ways, namely, mRNA cleavage, translation inhibition, and expression silencing mechanism (**Figure 1**) (Zhang et al., 2022b). Another type of ncRNAs includes the lncRNAs, which are longer than 200 nt in length and lack a coding sequence or an open reading frame (ORF) (Wang et al., 2015; Rai et al., 2019). They are classified into three types based on their genomic location: intronic lncRNAs, antisense lncRNAs, and long intergenic ncRNAs (**Figure 2A**) (Ma et al., 2013; Fan et al., 2015). LncRNAs regulate plant development *via* affecting protein–protein interactions, interacting with miRNAs, and encoding small peptides, among others (**Figures 2B–I**) (Sun and Lin, 2019; Fan et al., 2020; Wierzbicki et al., 2021). Phased small interfering RNAs (phasiRNAs) are 21- or 24-nt sncRNAs that are produced in a phased pattern from the *PHAS* locus and are typically triggered by miRNAs (Fei et al., 2013). Intriguingly, phasiRNAs are specifically expressed in the reproductive organs and are associated with anther development and male fertility in plants, but their mechanism of function is not very clear (Xia et al., 2019).

A large number of ncRNAs have been elucidated as regulators of the transcription factors (TFs) or protein-coding genes that participate in the formation of male sterility (Mishra and Bohra, 2018; Dong et al., 2022; Yan and Ham, 2022). Their mechanisms of action are either dependent on or independent of plant hormones, which ultimately affect the degeneration of the tapetum, formation of microspores, and the release of mature pollen grains (Wei et al., 2015; Lei and Liu, 2020; Bohra et al., 2021; Han et al., 2021; Li et al., 2021). How do ncRNAs regulate the male-sterile phenotypes in plants? The functions of the new ncRNAs related to male sterility are summarized in this review.

## MiRNAs as key players in plant male sterility formation

### MiRNAs participate in male sterility through regulating plant hormone homeostasis

**Figure 3:**
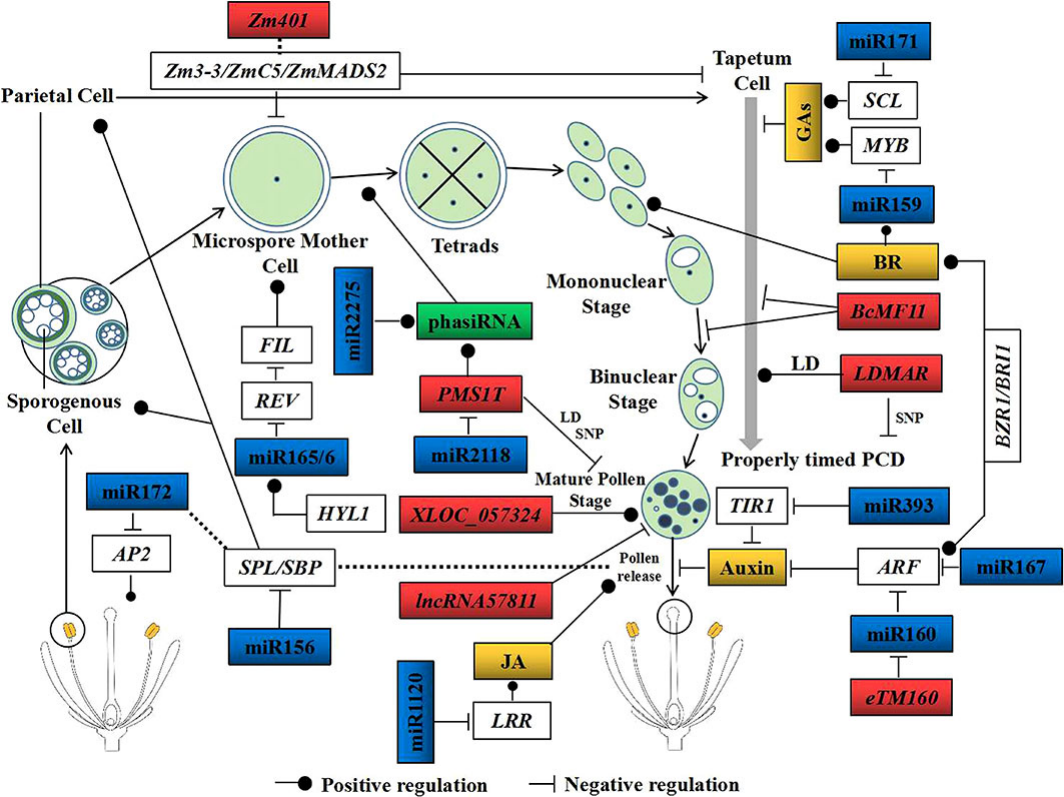
Critical non-coding RNAs (ncRNAs) participate in male organ development. Male organ development consists of six stages: the sporogenous cell stage, microspore cell stage, tetrad stage, mononuclear stage, binuclear stage, and the mature pollen stage. The *blue rectangles* represent the miRNAs that participate in male sterility, the *red rectangles* indicate the long non-coding RNAs (lncRNAs) related to pollen development in crops, the *green rectangles* indicate the phased small interfering RNAs (phasiRNAs) found in crops, and the *yellow rectangles* indicate the plant hormones involved in male organ development. NcRNAs participate in the dependence or non-dependence of male sterility on hormones. Among them, miR160, miR167, miR393, miR171, miR159, miR1120, and *eTM160* regulate pollen development mediated by auxin, BRs, GAs, and JA. Others function by regulating the transcription factors or methylation levels. The *dashed line* denotes indirect regulation and control. *MYB*, MYB domain protein; *SCL*, scarecrow-like protein; *ARF*, auxin response factor; *TIR*, transport inhibitor response protein; *SPL*, SQUAMOSA promoter binding protein-like; *FIL*, filamentous flower; *HYL1*, hyponastic leaves 1; LD, long-day condition; SNP, single nucleotide polymorphism; *BRs*, brassinosteroids; *JA*, jasmonate; *GAs*, gibberellins.

Plant hormones are critical regulators of reproductive development, and inhibition of their synthesis and signal transduction will cause male sterility. Auxin was the first plant hormone to be recognized. The auxin signaling F-box (AFB) proteins, auxin response factors (ARFs), heat shock protein 90 (HSP90), and co-chaperone *SGT1* (suppressor of the G2/M allele of *skp1*) are important moderators of auxin signaling transduction (Tian et al., 2017). *ARF6* and *ARF8* regulate the expression patterns of the genes that respond to auxin through binding to their promoter elements (Nagpal et al., 2005). The *arf6* and *arf8* double loss-of-function mutants showed flower arrest, stamen shortening, and anther indehiscence to release pollen grains (Nagpal et al., 2005). *ARF17* is essential for pollen wall patterning in *Arabidopsis* through modulating primexine formation. The *arf17* mutant did not have primexine within tetrads and exhibited a male-sterile phenotype (Yang et al., 2013). miRNAs, which target *ARF*, *AP2*, *AFB2*, *HD-ZIP*, and *TIR*, among others, regulate hormone metabolism and cause the expression of the pollen development genes to change, ultimately leading to male sterility (**Figure 3**) (Nie et al., 2018). Expression and function analysis revealed that the miR160/*ARF16*, miR393a/*TIR1*, and miR3444a/*F-box* interaction events participate in auxin signaling transduction and are important to CMS formation in radish (Zhang et al., 2016a). The same results were also found in male-sterile lines in cotton (Wei et al., 2013; Nie et al., 2018). Comparison of the small RNA expression profiles of the CMS line UPAS 120A and the isogenic fertile line UPAS 120B revealed 316 miRNAs and 961 target protein-coding genes, which include a variety of TFs, play key roles in plant reproduction, such as auxin and ethylene response factors. This result indicates that the signaling pathways activated by abscisic acid (ABA), auxin, etc., are important to the formation of male sterility (Bohra et al., 2021).

Both miR160 and miR167 interact with ARFs and participate in the auxin signaling pathway (Wu et al., 2006; Ding et al., 2017). In high temperature (HT)-insensitive (84021) and HT-sensitive (H05) cotton cultivars, miR160 targeted *ARF10*, *ARF16*, and *ARF17* (Ding et al., 2017). Overexpression of miR160 in cotton (e.g., in the miR160 OE2 line) resulted in higher miR160 and lower *ARF10* and *ARF17* expression, and the miR160 OE2 line was fertile, similar to the null control under normal temperature condition (Ding et al., 2017). However, after HT treatment, only the miR160 OE2 line showed anther indehiscence. Therefore, the overexpression of miR160 in cotton inhibited the expression levels of *ARF10* and *ARF17* and then activated auxin signaling, ultimately leading to male sterility under HT stress (Ding et al., 2017). Interestingly, the biosynthesis of jasmonic acid (JA) was suppressed in the anthers of the miR160 OE2 line under HT stress (Ding et al., 2017). In contrast to miR160, the overexpression of miR157 in cotton disrupted the signal transduction of auxin in ovules and young buds, generating indehiscent anthers and sterile pollen grains in the HT condition (Liu et al., 2017). miR167 has also been found to regulate the *ARF* TFs and cleave the *ARF6* transcripts *in vivo* (Wu et al., 2006). *mARF6* was obtained from the introduction of eight mutations into the miR167 target sites in the coding sequence of *ARF6*. When *mARF6* was overexpressed in *Arabidopsis*, transgenic plants with higher *ARF6* protein abundance and their flowers were sterile. Transforming the *ARF6* overexpression vector into the wild-type *Arabidopsis*, although the *ARF6* transgenic plants had *ARF6* levels similar to or higher than those of *mARF6* transgenic plants, the *ARF6* transgenic plants showed fertile flowers (Wu et al., 2006). These results indicate that the loss of miR167/*ARF6* interactions caused abortive pollen and that miR167 is essential for normal fertility in *Arabidopsis*. TamiR167a was upregulated in anthers during the pollen development processes in the photo-thermosensitive genic male-sterile (PTGMS) wheat line BS366, while its target *TaARF8* was downregulated under sterile conditions (Wang et al., 2019a). The overexpression of miR167a in *Arabidopsis* resulted in the downregulation of both *AtARF6* and *AtARF8*. Transgenic *Arabidopsis* showed fewer contents of endogenous JA and indole acetic acid (IAA), and their flowers were male sterile (Wang et al., 2019a).

The brassinosteroids (BRs) are a group of steroid hormones isolated from rape pollen that have high biological activity (Li and He, 2020; Nolan et al., 2020). BRASSINOSTEROID INSENSITIVE 1 (*BRI1*) is the receptor of BRs, and *BES1*/*BZR1* are essential TFs in the BR signaling pathway (Yan et al., 2020). Although auxin and BR are two different hormones, their signaling transduction pathways interact and together regulate a variety of developmental processes in plants (Zakharova et al., 2022). *BZR1* interacts with ARF proteins to directly target multiple auxin signaling components. In addition, ARFs could bind to the promoter of *BRI1* and then activate BR signaling (**Figure 3**) (Tian et al., 2017). The BR biosynthesis mutant *cpd*, the responsive mutant *bril-116*, and the signal transduction mutants *bin2-1* and *qui-1* all presented male-sterile phenotypes (Ye et al., 2010; Chen et al., 2019). Leucine-rich repeat receptor-like kinase (LRR-RLK) interacts with *BRI1* and modulates BR signaling. It plays an important role in plant developmental and physiological processes (Zhao et al., 2002). Some researchers have proven that LRR-RLKs participate in the differentiation of microsporocytes and tapetum cells, which eventually led to male sterility (Zhao et al., 2002). In the wheat thermosensitive cytoplasmic male-sterile line YS3038, *TaeRPK* (a LRR-RLK gene) was targeted by unconservative_chr3B_part2_17629 (belonging to the MIR9657 family) and was upregulated under sterile conditions compared to the fertile conditions. Silencing of *TaeRPK* using the VIGS (virus-induced gene silencing) method in YS3038 resulted in the seed setting rates being significantly lower than those of the negative control plants (Han et al., 2021). In general, gibberellins (GAs) and BRs play similar roles in plant developmental processes (Barro-Trastoy et al., 2020). BRs regulate the expression of miR159d and its target gene *OsGAMYBL2* in rice (Gao et al., 2018). Furthermore, *OsGAMYBL2* coordinates BR signaling and GA biosynthesis by regulating the expression of the BR signaling gene *BU1* and two GA biosynthetic genes, *CPS1* and *GA3ox2* (Gao et al., 2018). GAs also participate in the formation of male sterility. The overexpression of *gai* (a GA signal transduction gene) using the reproductive tissue-specific promoter in *Nicotiana tabacum* and *Arabidopsis* resulted in the abortion of the male genital organ (Huang et al., 2003).

In addition, miRNAs and their targets mediate a complex regulatory network associated with the metabolism of auxin, cytokinin, ABA, and GAs and play important roles in cell division and flower induction in apple. Among them, miR396, miR160, and miR393 and their target genes are related to auxin, while miR159, miR319, and miR164 and their targets are involved in ABA and GA biosynthesis and signal transduction, eventually regulating bud growth and flower bud formation (Xing et al., 2016). The interaction events of miR172/*AP2*, miR156/*SPL*, and miR171/*SCL* participate in the GA/ABA and GA/auxin signaling pathways, further regulating the flowering time. Moreover, miR825 and miR1120 interact with their respective target genes (*CaBP* and *LRR*) to modulate pollen development *via* the CRY/PHY and JA signaling pathways (Bai et al., 2017).

### Conserved miRNAs cause plant male sterility independent of hormones

Several miRNAs appear to be universally expressed and conserved among diverse angiosperms, while a small number is also conserved in bryophytes, lycopods, and gymnosperms (Axtell and Bowman, 2008). Conservative miRNAs are known to function in diverse development processes, and a proportion of them has been reported to participate in the development of male sterility. Research studies in cotton CMS have demonstrated that miR399, miR172, and miR393 were differentially expressed between wild-type and male-sterile anthers (Wei et al., 2013; Nie et al., 2018). Similar results have also been found in the soybean CMS line NJCMS1A and the wild-type line NJCMS1B (Ding et al., 2016b). To date, at least nine conserved miRNA families have been reported to regulate the male organ development, namely, miR156, miR159, miR160, miR164, miR166/165, miR167, miR169, miR172, and miR319 (Luo et al., 2013b; Spanudakis and Jackson, 2014; Sun et al., 2021). These miRNAs target various protein-coding genes and are involved in flower development processes independently of plant hormones (**Figure 3**).

miR156 is conserved in all angiosperms studied thus far, and its mature sequences from 40 species showed 75% similarity, such as in *Arabidopsis*, *Gossypium hirsutum*, *N. tabacum*, *Oryza sativa*, *Triticum aestivum*, and *Zea mays* (Sunkar and Jagadeeswaran, 2008). miR156 regulated 142 protein-coding genes, including three ATPase family AAA domain-containing proteins (e.g., *ATD1A*), four SQUAMOSA promoter binding protein-like (*SPL*), two calcium-transporting ATPases (*ACA1* and *ACA2*), two cyclin-dependent kinases (e.g., *CKB22*), and a floral homeotic protein (*MADS2*) (Qu et al., 2021). Research in the soybean CMS line NJCMS1A and its restorer line NJCMS1C provided proof that the expression of miR156b is more than 1.5-fold greater in NJCMS1A, and the expression patterns of miR156b and *SPL9* showed opposing trends in early-stage flower buds (**Figure 3**) (Ding et al., 2019). *SPL* interacted with *FT* (FLOWERING LOCUS T) and affected the flowering metabolic pathway (Schmid et al., 2003). In *Arabidopsis* seedlings, a higher miR156 and a lower *SPL* expression prevented precocious flowering. Subsequently, miR156 was downregulated and *SPL* was upregulated, which provided a permissive environment for flowering (Wang et al., 2009). miR156 targets all *SPL* genes, except for *SPL8*, and silencing the *SPL8* gene caused a semi-sterile phenotype in *Arabidopsis*. Furthermore, the overexpression of miR156 in the *spl8* mutant resulted in fully sterile plants, while overexpression of the miR156-resistant *SPL* could restore fertility of the *spl8* mutant (Xing et al., 2011). In addition, miR172 was regulated by miR156 through *SPL9*, and the overexpression of miR172 in *Arabidopsis* promoted plant flowering (**Figure 3**) (Chen, 2004; Jung et al., 2007).

Members of the miR159 family have been found in *Arabidopsis*, *O. sativa*, *T. aestivum*, *Z. mays*, and other angiosperms with more than 80% sequence similarity. Their target genes are also conserved across different plant families (Luo et al., 2013b; Guo et al., 2021). In rice, miR159 and its putative targets (*OsGAMYB* and *OsGAMYBL1*) were co-expressed in reproductive organs, and the miR159-mediated *OsGAMYB* and *OsGAMYBL1* mRNAs were degraded in anthers (**Figure 3**) (Tsuji et al., 2006). *GAMYB* has been proven to participate in flower development in many plants (Millar and Gubler, 2005). Overexpression of miR159a in rice inhibited the expression patterns of *OsGAMYB* and *OsGAMYBL1*, which finally led to abnormal flowers and a male-sterile phenotype (Tsuji et al., 2006). Moreover, *GAMYB*-like was also regulated by miR159 in *Arabidopsis*, wherein its overexpression led to the downregulation of *AtMYB33* and caused anther defects and male sterility (Millar and Gubler, 2005).

Members of the miR164 family are completely identical among 28 species, and they are potentially capable of targeting the *NAC* TFs, including CUP-SHAPED COTYLEDON1 (*CUC1*) and *CUC2* in *Arabidopsis* and *EgNAM1* and *PdNAM1* in palms (Luo et al., 2013b). miR164 was upregulated in pollen at the later developmental stages in sterile wheat plants, while *CUC*/*NAM* was downregulated in the sterile group, particularly in the later anther development stages (Bai et al., 2017). Osa-miR164d had higher expression in the meiosis stage of the photo-thermosensitive genic male-sterile (PTGMS) rice line WXS and showed negative correlation with its target, *NAM*. The pollen meiosis stage is important for the fertility transition of the WXS line, and the upregulation of osa-miR164d in this stage indicated that miR164d is critical to pollen development (Zhang et al., 2016b).

miR165/6 are involved in sporangium, microspore mother cell, and microspore development. Their mature sequences have 90% similarity among 36 species, and their target genes [Class III HOEMEODOMAIN LEUCINE ZIPPER (*HD-ZIP III*) TFs] are highly conserved in land plants (Luo et al., 2013b). REVOLUTA (*REV*) is a member of *HD-ZIP III* that has shown negative correlation with FILAMENTOUS FLOWER (*FIL*). *REV* regulates the establishment of anther polarity, while *FIL* is critical to the development of sporangium. HYPONASTIC LEAVES1 (*HYL1*) participates in miRNA biogenesis and plays a critical role in establishing the stamen architecture in *Arabidopsis*. *HYL1* deficiency was shown to reduce the accumulation of miR165/6 and to increase the expression level of *REV* in anthers (Wu et al., 2007; Lian et al., 2013), further resulting in a decreased sporangium and even male infertility (**Figure 3**) (Lian et al., 2013).

## MiRNA acts as a key factor in the miRNA–lncRNA–mRNA interaction networks

### MiRNA and lncRNA interact to regulate auxin metabolism and affect male sterility

Plant lncRNAs act as endogenous target mimics (eTMs) of miRNAs (**Figures 2D** and **3**). Based on their incomplete binding to the miRNA seed regions, lncRNAs could block the interaction between the miRNAs and their authentic targets (Yoon et al., 2014; Meng et al., 2021). The lncRNA *eTM160* acted as the eTM of miR160 and was specifically expressed in rice anthers during the meiotic and young microspore stages (Wang et al., 2017). miR160 targets *ARF18*, a gene that encodes an ARF and, as a transcriptional regulator, mediates auxin responses (Liu et al., 2015). When *eTM160* (OE-eTM160) and the *eTM160* mutant (OE-eTM160M) were overexpressed in rice, it was found that miR160 was downregulated, but *ARF18* was upregulated, in OE-eTM160 plants; however, this phenomenon did not exist in OE-eTM160M plants. These results indicate that *eTM160* blocked the binding of miR160 to *ARF18* and overexpressed *eTM160* in rice, resulting in the upregulation of *ARF18*. It is worth noting that the lemma and palea of OE-eTM160 failed to open and showed abnormal fertility during the reproductive development stage; a similar phenomenon also occurred in rice with overexpressed *ARF18* (Wang et al., 2017). In *B. rapa*, the lncRNA *bra-eTM160-2*, which acted as the target mimic of miR160, was specifically and predominantly expressed in inflorescence. The overexpression of *bra-eTM160-2* in *B. rapa* (OE-bra-eTM160-2) led to bra-miR160-5p being significantly decreased in inflorescence, while the target genes of miR160, including *BrARF10*, *BrARF16-1*, *BrARF16-2*, *BrARF17-1*, and *BrARF17-2*, were significantly increased. OE-bra-eTM160-2 plants possessed small and curled rosette leaves and showed symptoms of auxin deficiency. Although the floral organs of OE-bra-eTM160-2 appeared normal, the anthers started aborting from the uninucleate microspore stage and the microspores were hollow and collapsed, which finally caused the pollen grains to be small, shrunken, and without viability (Huang et al., 2018).

Based on transcriptome sequencing during anther development in the CMS-D_2_ line A, maintainer line B, and restorer-of-fertility line R in cotton, 328 and 431 differentially expressed lncRNAs were identified as unique in the A–B and A–R comparisons, respectively. *TCONS_00807084*, which was predicted to be a putative precursor of miR160b, showed higher abundance in the A line than in both the B and R lines. miR160b targeted *GhARF17*; therefore, it was speculated that miR160b and *TCONS_00807084* might regulate anther development together through influencing the auxin metabolic pathway (**Figure 3**). In addition, based on the analysis of the putative miRNA–lncRNA–mRNA regulatory network, it was found that the interaction events, such as csi-miR160 negatively regulating *Gh_D06G0360* and *TCONS_01087328* and *TCONS_00675896* positively regulating *Gh_D02G0666*, were important to CMS and fertility restoration through their involvement in auxin and GA metabolic processes in cotton (Zhang et al., 2019).

In summary, the lncRNA–miR160–*ARF* interaction networks might participate in pollen development by participating in auxin regulation in many plants.

### MiRNA–lncRNA participate in male sterility independent of hormones

Most plant miRNAs are generated from long non-coding precursors (Bielewicz et al., 2013), with similar results also found in animals (Raini et al., 2007). Generally, miRNAs are produced by the introns of lncRNAs, which suggests that the donor lncRNAs not only act as pre-miRNAs but also have additional roles mediated by exons (Bielewicz et al., 2013). In the rice photoperiod-sensitive male-sterile line Nongken 58S, *LDMAR* (long-day-specific male fertility-associated RNA) was important in pollen abortion. It could be processed into a 136-nt intermediate precursor firstly, then produce a 21-nt small RNA for function (Zhu and Deng, 2012). In addition, sufficient abundance of complete *LDMAR* transcripts is also important for pollen to develop normally under long-day conditions. Elevated methylation levels inhibited the transcription of *LDMAR*, specifically under long-day conditions, then led to abnormal degeneration of the tapetum due to premature programmed cell death (PCD), eventually causing photoperiod-sensitive male sterility (Ding et al., 2012). Analysis of the lncRNA expression profiles in the cotton CMS and the restorer (Rf) and maintainer line flower buds found that 1,531 lncRNAs and parts of them showed significant differential expression patterns between these three lines. Among them, 94 lncRNAs were identified as putative precursors of 49 miRNAs, indicating that the miRNA/lncRNA interaction networks probably function as a complex regulatory pathway during pollen development (Hamid et al., 2020).

## Tissue-specific lncRNAs impact the occurrence of male sterility independently

Aside from interacting with miRNAs, lncRNAs regulate plant development through multifarious ways. In recent years, more and more reproduction organ-specific lncRNAs have been identified, and the number has surpassed that of protein-coding transcripts (Yang et al., 2020). These findings indicate the great potential of plant lncRNAs to function specifically in reproductive organs.

*FSNR* is a unique lncRNA identified from the MT genomes in the CMS line. It presented much higher expression and more edited sites in females than in hermaphrodites (Stone et al., 2017). Short open reading frame mRNA (sORF-mRNA) refers to the mRNA containing only short ORFs and encoding less than 100 amino acids. It is usually expressed in specific development stages (MacIntosh et al., 2001). *Zm401* is a sORF-mRNA in maize that is specifically expressed in tapetal cells and in the microspores of pollen grains (Ma et al., 2008). The protein-coding genes *Zm3-3*, *ZmC5*, and *ZmMADS2* have been proven to be important in pollen development. Silencing *Zm401* in maize altered the expression patterns of *ZmMADS2*, *Zm3-3*, and *ZmC5* and caused abnormal microspore and tapetum development (**Figure 3**) (Ma et al., 2008). *BcMF11*, a novel pollen-specific lncRNA from Chinese cabbage, delayed the degradation of the tapetum, caused asynchronous separation of the microspore, and aborted the development of pollen grains (**Figure 3**) (Song et al., 2013). Through strand-specific RNA sequencing (ssRNA-seq), Zhang et al. (2014) found that the lncRNA *XLOC_057324* was specifically expressed in young panicles and pistils. Transfer DNA (T-DNA) insertion decreased the abundance of *XLOC_057324* in rice, and the mutants were flowering earlier and were male sterile (**Figure 3**) (Zhang et al., 2014). A total of 444 differentially expressed lncRNAs were detected in rice anthers. Among them, *TCONS_00057811* showed anther specificity and had the highest abundance during the meiosis stage (**Figure 3**) (Li et al., 2020). The overexpression of *TCONS_00057811* in rice altered the expression pattern of *LOC_Os12g41350* (Meiotic Asynaptic Mutant 1), a meiosis-related gene, which resulted in lower pollen fertility (29.70%) and seed setting (33%) than in control plants (Li et al., 2020). Interestingly, all of the aforementioned lncRNAs (**Figure 3**), which participated in the formation of male sterility through interacting with miRNAs, also presented tissue-specific expression. Apart from the well-studied lncRNAs, some lncRNAs also participate in male sterility, but with unknown mechanisms (**Table 1**).

**Table 1:** Long non-coding RNAs (lncRNAs) involved in plant reproductive development.

Name	Length(nt)	Species	Mode of action	Function	CMS/GMS	Reference
*COOLAIR*	–	*Arabidopsis*	Chromatin modification	Flowering timing	–	Csorba et al., 2014
*COLDAIR*	1,156	*Arabidopsis*	Chromatin modification	Flowering timing	–	Heo and Sung, 2011
*BcMF11*	828	*Brassica campestris*	Unknown	Pollen development	GMS	Song et al., 2013
*eTM160-1/eTM160-2*	530/464	*Brassica rapa*	eTM of miR160	Pollen development	GMS	Huang et al., 2018
*CsM10*	208	*Cucumis sativus*	Unknown	Male sex differentiation	GMS	Cho et al., 2006
*TCONS_00807084*	718	*Gossypium hirsutum*	eTM of miR160	Anthers development	CMS	Zhang et al., 2019
*eTM160*	688	*Oryza sativa*	eTM of miR160	Anthers development	GMS	Wang et al., 2017
*PMS1T*	1,453	*Oryza sativa*	Target of miRNA	Anthers development	GMS	Fan et al., 2016
*LDMAR*	1,236	*Oryza sativa*	Methylation/precursor of sRNA	Anther development	GMS	Ding et al., 2012; Zhu and Deng, 2012
*XLOC_057324*	758	*Oryza sativa*	Unknown	Panicle development	GMS	Zhang et al., 2014
*TCONS_00057811*	635	*Oryza sativa*	Interaction with mRNA	Pollen development	–	Li et al., 2020
*FSNR*	–	*Silene vulgaris*	Unknown	Gamete transition	CMS	Stone et al., 2017
*Zm401*	1,200	*Zea mays*	Interaction with mRNA	Microspore and tapetum development	GMS	Ma et al., 2008

eTM, endogenous target mimic; CMS, cytoplasmic male sterility; GMS, genic male sterility.

## PhasiRNAs play important regulatory roles in plant male sterility

Apart from being processed into miRNAs, lncRNAs have been identified as putative targets of miRNAs. After cleavage, lncRNAs form small intermediate RNAs to function. Similar to *LDMAR*, *PMS1T* (photoperiod-sensitive genic male sterility 1) was also specifically more highly expressed in young panicles in the rice photoperiod-sensitive male-sterile line (Fan et al., 2016). miR2118 is conserved in plants and is critical for inducing 21-nt phasiRNAs through the cleavage of lncRNAs. *PMS1T* is a target of miR2118 and could produce 21-nt-dominated phasiRNAs after cleavage. A sufficient number of phasiRNAs in panicles is necessary for the formation of male sterility under long-day conditions, but the mechanism is not well understood. A single nucleotide polymorphism (SNP) nearby the miR2118 target sites of *PMS1T* inhibited the action of miR2118 and reduced the abundance of phasiRNAs. A lower number of phasiRNAs eventually restored the male fertility in rice under long-day conditions (Fan et al., 2016).

In addition to the aforementioned miR2118–*PMS1T*–phasiRNA–target regulatory cascades, loss of miR2118 in rice was also demonstrated by Araki et al. (2020) to cause severe male and female sterility since the number of miR2118-dependent 21-nt phasiRNAs significantly decreased in the anther wall from stage 1 to stage 6. Through sncRNA and degradome sequencing of the developing flowers in the male-sterile cybrid pummelo (G1+HBP) and its fertile parent Hirado Buntan pummelo (HBP), a total of 2,235 unique 21-nt phasiRNAs were identified, with 88 of them showing significant expression differences in developing flowers between G1+HBP and HBP. Of these 88 phasiRNAs, 34 were generated from the protein-coding genes, while the others were produced from lncRNAs (Fang et al., 2020). In a derivative line of the male-sterile outer cell layer 4 (*ocl4*) mutant of maize, the biogenesis of the 21-nt phasiRNAs was largely dependent on *Ocl4*, and it was mediated by miR2118 (Yadava et al., 2020). Further research found that the accumulation levels and the fractions of phasiRNAs were the highest in the wild type, as expected, but were also significantly higher in the conditionally fertile *ocl4* compared to the *ocl4* sterile samples, suggesting partial restoration of the phasiRNAs in the conditionally fertile plants (Yadava et al., 2020). On the other hand, miR2118, miR390, miR472, miR3954, miR158, and miR482 could trigger the production of 21-nt phasiRNAs (Fang et al., 2020; Tripathi et al., 2021). miR2275 is the unique miRNA known to trigger the production of 24-nt-dominated phasiRNAs, and all of these trigger events preferentially occurred in the tapetum and meiocytes. miR2275 is specifically expressed in the tapetum during the early meiosis stage, and its expression is quickly reduced in other stages of male gametogenesis in both rice and maize (Tamim et al., 2018). In maize, the tapetum is the primary site of the 24-PHAS precursor and *Dcl5* transcripts, as well as the resulting 24-nt phasiRNAs, and then the phasiRNAs are mobile from the tapetum to meiocytes and to other somatic cells (Zhou et al., 2022). Generally, maize male-sterile mutants with subepidermal defects lack 24-nt phasiRNAs (Zhai et al., 2015). Male fertility in maize requires all four basic helix–loop–helix genes, including *Ms23*, *Ms32*, basic helix–loop–helix 122 (*bHLH122*), and *bHLH51*. Interestingly, the male-sterile *ms23*, *ms32*, and *bhlh122-1* mutants lack 24-nt phasiRNAs and the precursor transcripts from the corresponding 24-PHAS loci, while the *bhlh51-1* mutant has wild-type levels of both precursors and small RNA products, suggesting that *bHLH* TFs regulate male sterility by directly binding to their target promoters or through their indirect regulation of the downstream TFs dependent on phasiRNAs (Nan et al., 2022).

## Plant mitochondrial genes and the involved CMS mechanism

Although ncRNAs play critical roles in male sterility formation, most of them are related to GMS. CMS is generally caused by the uncoordinated interactions between the MT and nuclear genomes (Bohra et al., 2016; Nie et al., 2020; Takatsuka et al., 2022). Plant MT genomes are dynamic genomes, the sequences of which frequently undergo rearrangement, recombination, structural variation, and exogenous or internal sequence migration (Chen et al., 2017). Previous studies have shown that CMS-associated genes were produced by dynamic changes in the MT genomes (Chen et al., 2017; Kim et al., 2019). To date, more than 30 CMS-related MT genes have been identified in different crop species (Kim and Zhang, 2018), including o*rfH79* in CMS-HL (Wang et al., 2013; Ding et al., 2016a), *orf352* in CMS-RT102 (Okazaki et al., 2013), and *orf138* in CMS-O (Bellaoui et al., 1998). Most of these genes have the following characteristics: 1) are located nearby the MT functional genes; 2) are associated and co-transcribed with the essential MT genes; 3) have chimeric ORFs; and 4) possess transmembrane domains (Chen et al., 2017; Li et al., 2018). *Orf79* encodes a protein that includes 79 amino acids and contains an N-terminus with similarity to COX1 in cytoplasmic male-sterile rice. It is also co-transcribed with the upstream gene *atp6* (Kazama et al., 2016). In CMS-T maize, compelling evidence has indicated that the MT gene *T-urf13* (ORF 13 unique to the T cytoplasm of maize) is critical to CMS formation. *T-urf13* is a unique chimeric sequence that comprises part of *atp6* and encodes a nonessential amino acid (URF13) with an inner MT membrane domain (Dewey et al., 1987). In petunia CMS, *pcf* is located close to two MT genes, *nad3* and *rps72*, and contains part of the coding region of *atp9* and part of each exon of *cox11*, the same as *T-urf13* (Young and Hanson, 1987).

**Figure 4:**
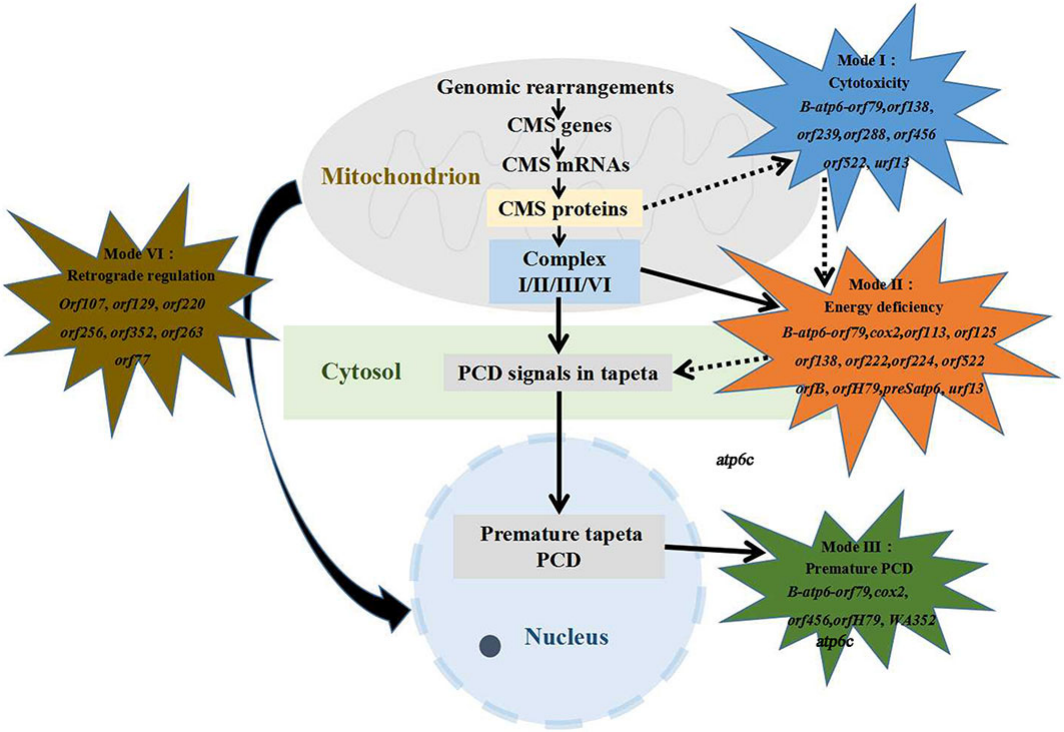
Four function mechanisms of cytoplasmic male sterility (CMS) genes. More than 30 mitochondrial genes have been found to participate in CMS formation, with several genes possibly possessing multiple mechanisms of action affecting male sterility. Some of the genes involved in CMS formation have unknown mechanisms. *Dotted arrows* represent the potential co-action between two modes.

Generally, four critical models of MT genes mediate CMS (**Figure 4**), and each CMS gene causes male sterility possibly through multiple mechanisms (Chen et al., 2017). Pollen development is a highly energy-consuming process, and a large number of research studies have indicated that CMS may be triggered by abnormal mitochondria that failed to provide enough energy for rapid cell differentiation (Chen and Liu, 2014). In the Honglian CMS system of rice, the MT protein ORFH79 interacted with the complex III QCR10 subunit homologous protein P61 and inhibited the activity of complex III, then causing male sterility due to the decrease in energy and the increase in ROS content (Wang et al., 2013). A properly timed PCD of the tapetum provides nutrients for pollen maturation; otherwise, a premature or delayed PCD leads to pollen abortion (Twell, 2011; Xie et al., 2022). In the wild-abortive CMS (CMS-WA) system of rice, WA352 interacted with the nuclear-encoded MT protein COX11, destroyed the function of COX11 in peroxide metabolism, and triggered premature tapetal PCD, consequently leading to pollen abortion (Luo et al., 2013a). The MT gene *atp6c* conferred male sterility in CMS-C maize, and it more strongly interacted with ATP8 and ATP9 than with ATP6 during the assembly of the F1Fo-ATP synthase (F-type ATP synthase, ATPase), thereby causing a reduction of the quantity and activity of F1Fo-ATP synthase. The reduced F1Fo-ATP synthase activity caused the accumulation of excess protons in the inner membrane space of the mitochondria, triggering a burst of ROS, premature PCD of the tapetal cells, and the abortion of pollen (Yang et al., 2022). Confirmed evidence from transgenic experiments has provided proof that the maize MT gene *urf13* is toxic to cell viability (Levings, 1993). Retrograde regulation refers to the phenomenon of nuclear genes being regulated by organelle genes (Fujii et al., 2007). In the rice CMS-CW (rice hybrids obtained reciprocally between cultivated and wild varieties) system, MT genes acted as regulators of some nuclear genes that are preferentially expressed in male-sterile lines and related to alternative oxidase; the upregulation of these nuclear genes in anthers resulted in male sterility (Fujii et al., 2007). *ZmDREB1.7*, which showed higher expression in the sterile microspores in maize, activated the CMS-related MT gene *orf355* and caused male sterility. Interestingly, *orf355* also upregulated the expression pattern of *ZmDREB1.7* through activating MT retrograde signaling. These results indicate that the retrograde regulation pattern between the nuclear regulator and the mitochondrial CMS gene plays a critical role in the formation of male sterility in maize (Xiao et al., 2020).

## Conclusion and future perspectives

Male sterility is a crucial attribute in the production of plant hybrids. To date, many ncRNAs have been proven to function in the formation of male sterility; however, most of them are related to GMS. To explore the mechanism of CMS, it is absolutely imperative to determine the large-scale ncRNAs encoded by the cytoplasmic genome and to reveal their roles in chimeric cytoplasmic genes. NcRNAs target hormone-responsive TFs and regulate plant male organ development by affecting hormone homeostasis, such as auxin, BRs, GAs, ABA, and JA (Xing et al., 2016; Bai et al., 2017). However, the development of male-sterile lines using exogenous hormone analogues is both an opportunity and a challenge. The rapid development of sequencing techniques has made it easier to discover ncRNAs (Li, 2020), and the CRISPR/Cas9 editing system has been used in the development of male sterility in plants (Jiang et al., 2021). Advancements in technology have offered new opportunities to apply the CRISPR/Cas9 system to reveal the functions of ncRNA mutants (Goyal et al., 2017; Wang et al., 2019b), which will be beneficial for exploring the mechanisms of ncRNAs in male sterility and will be conducive to creating new sterile lines using the transgenic method.

## Author contributions

All authors listed have made direct contributions to the work and approved it for publication. JH, HN, CC, and HL wrote parts of the manuscript. JH, HN, and JK contributed to language revision and figure design. All authors contributed to the article and approved the submitted version.
